# Nitidine chloride induces caspase 3/GSDME-dependent pyroptosis by inhibting PI3K/Akt pathway in lung cancer

**DOI:** 10.1186/s13020-022-00671-y

**Published:** 2022-09-29

**Authors:** Fei Yu, Weidan Tan, Zhiquan Chen, Xiaoju Shen, Xiaoxiang Mo, Xiaocheng Mo, Jingchuan He, Zhihua Deng, Jie Wang, Zhuo Luo, Jie Yang

**Affiliations:** Department of Pharmacology, School of Pharmacy, 406 Graduate School of Guangxi Medical University, Nanning, 530021 Guangxi People’s Republic of China

**Keywords:** Nitidine chloride, Lung cancer, Pytoptosis, GSDME, PI3K/Akt

## Abstract

**Background:**

As the increasing mortality and incidence of lung cancer (LC), there is an urgent need to discover novel treatment agent. In this study, we aimed to investigate the anti-LC effects of nitidine chloride (NC), a small molecular compound extracted from Chinese herbal medicine, while detailing its underlying mechanisms.

**Methods:**

Cell viability was detected by MTT assays and five cell death inhibitors, including ferrostatin-1 (Fer-1), Z-VAD-FMK, necrostatin-1 (Nec-1), disulfiram (DSF) and IM-54 were used to explore the type of cell death induced by NC. The microscopic features of NC-induced pyroptosis were assessed by transmission electron microscopy (TEM) and the pyroptotic-related proteins such as caspase and gasdermin family, were examined by western blot. Network pharmacology was employed to predict the potential mechanisms of NC in lung cancer treatment. CETSA and DARTs were used to determine the activity of NC binding to targeted protein. Xenograft mice model was established to further investigate the inhibitory effect and mechanism of NC against LC.

**Results:**

The pyroptosis inhibitor (DSF) and apoptosis inhibitor (Z-VAD-FMK) but not IM-54, necrostatin-1, or Ferrostatin-1 rescued NC-induced cell death. Morphologically, H1688 and A549 cells treated with NC showed notably pyroptotic features, such as cell swelling and large bubbles emerging from the plasma membrane. Gasdermin E (GSDME) rather than GSDMC or GSDMD was cleaved in NC-treated H1688 and A549 cells with an increased cleavage of caspase 3. Combined with network pharmacology and molecule docking, PI3K/Akt signaling axis was predicted and was further verified by CETSA and DARTs assay. In addition, the activation of PI3K is able to rescue the pyroptosis induced by NC in vitro*.* In xenograft model of LC, NC significantly hindered the transduction of PI3K-AKT pathway, inducing pyroptosis of tumor.

**Conclusion:**

Our data indicated that NC is a potential therapeutic agent for the treatment of LC via triggering GSDME-dependent pyroptosis.

**Supplementary Information:**

The online version contains supplementary material available at 10.1186/s13020-022-00671-y.

## Introduction

Lung cancer (LC) is one of the most harmful malignant tumors worldwide [1]. Due to the complicated pathological classification of LC, the treatment strategies vary, such as surgical resection, radiotherapy, chemotherapy, targeted therapy and immunotherapy [2, 3]. Chemotherapy is the main treatment presently and cisplatin (DDP) is the representative chemotherapy drugs in the treatment of LC [4]. However rapid drug resistance and poor prognosis occur in patients with long treatment of DDP [5]. In addition, the severe toxic effect of DDP highly limits its therapeutic effect and application [6]. Therefore, there is an urgent need to develop a novel agent for LC therapy.

Nitidine chloride (NC) is a kind of benzophenanthridine alkaloid extracted from Chinese herbal medicine *Zanthoxylum nitidum (Roxb.) DC*, which has been used as a medicinal plant for thousands of years [7]. NC has been proposed as a potent anti-tumor agent in a variety of human cancers such as LC, breast cancer and liver cancer [8]. A plethora of mechanisms has been demonstrated in NC against cancer diseases such as inducing cell apoptosis [9], blocking cell cycle [10], inhibition of tumor invasion and migration [8, 11], impeding angiogenesis [33] and restraint of tumor stem cell-like characteristics [11], etc. Nevertheless, little is known about which type of cell death is triggered by NC in LC cells and the underlying mechanism remains to be elucidated.

In this study, we demonstrated that NC could induce caspase 3/GSDME-dependent pyroptosis in LC cells and transplantation tumors of mice. In addition, blockage of PI3K-Akt signaling was demonstrated to be involved in the pyroptosis triggered by NC.

## Materials and methods

### Chemiacals and materials

**Fig. 1:**
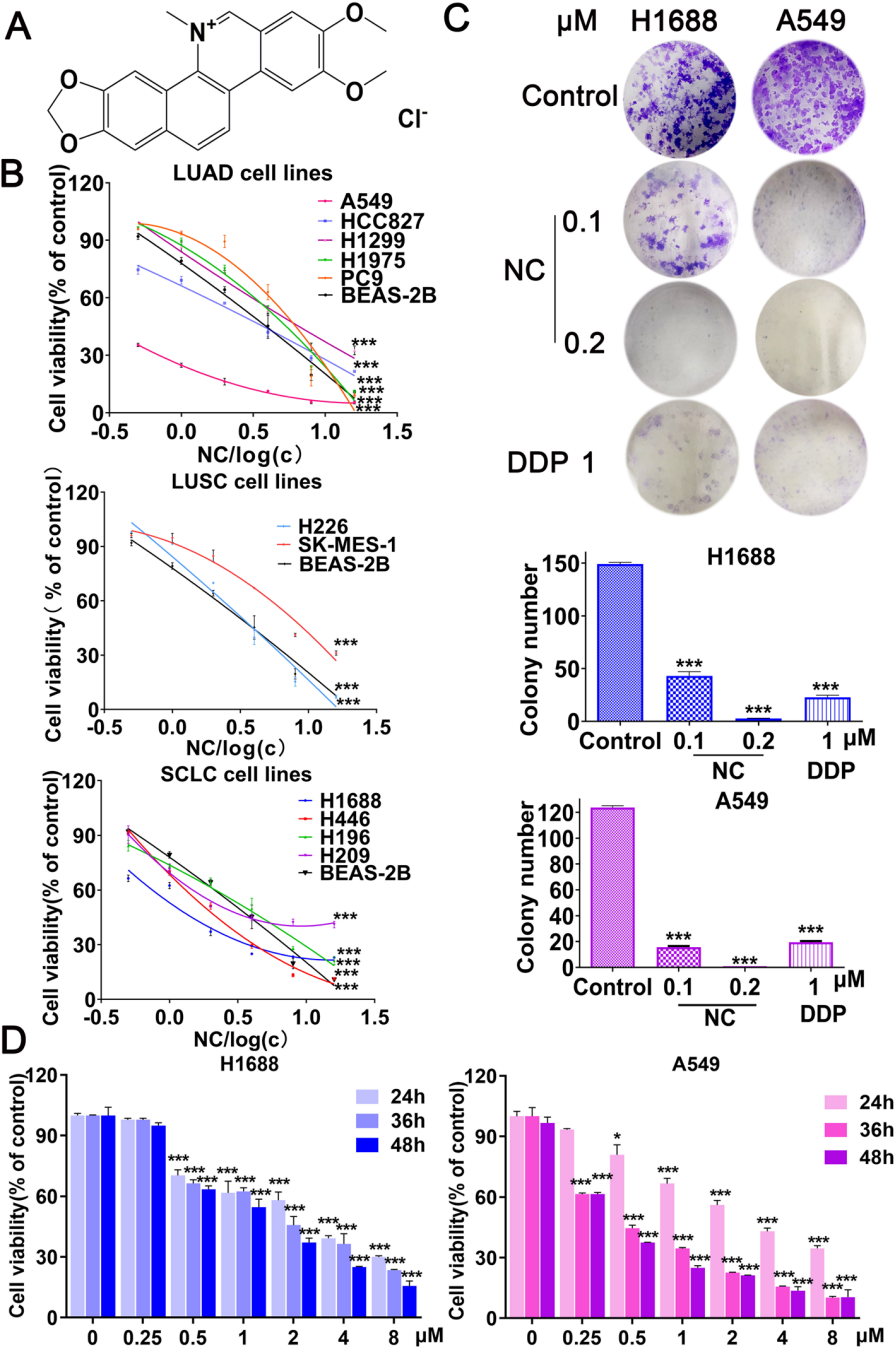
**A** Structure of NC. **B** The cell viability of normal lung cells BEAS-2B and 11 lung cancer cells are measured by MTT assay for 48 h. **C** Colony formation assays were performed using H1688 and A549 cells with or without NC or DDP for 10–14 days. **D** The cell viability of H1688 and A549 cells treated with the indicated concentrations of NC for 24, 36 and 48 h respectively. *P < 0.05, **P < 0.01, ***P < 0.001 *vs* control

NC (Fig. 1A) was purchased from Solarbio (Beijing, China), with the purity above 98%. DDP was purchased from Qilu Pharmaceutical Co., Ltd. (Shandong, China). Ferrostatin-1 (Fer-1), Z-VAD-FMK, Necrostatin-1 (Nec-1), Disulfiram (DSF), Z-DEVD-FMK and 740Y-P was purchased from MCE (America). 2-(1H-Indol-3-yl)-3-pentylamino-maleimide (IM-54) was purchased from Abcam.

### Cell lines and cell culture

NCI-H226, SK-MES-1, A549, NCI-HCC827, NCI-H1975, PC9 and BEAS-2B cells were purchased from Cell Bank of the Chinese Academy of Sciences (Shanghai, China). NCI-H209, NCI-H446, NCI-H1688 cells were purchased from national collection of authenticated cell cultures (Shanghai, China). NCI-H196 and NCI-H1299 cells were purchased from American Type Culture Collection (ATCC). All the culture media were supplemented with 10% fetal bovine serum (FBS, Biological Industries, Israel), penicillin (100 U/mL) and streptomycin (100 μg/mL, Solarbio, Beijing, China). The cells were incubated at 37 °C with 5% CO_2_.

### MTT (3- (4,5-dimethylthiazol-2-yl)-2,5-diphenyltetrazolium bromide) assays

MTT assays were performed as previously described [12]. Briefly, cells (3 × 10^3^ cells/mL) were plated in 96-well plates and treated with different concentrations of NC or DDP for 24, 36, and 48 h respectively. DMSO (0.1%) served as vehicle control. Cells were incubated with 20 μL of MTT (5 mg/ml) for 4 h to form purple formazan crystals. Then, purple formazan crystals were dissolved by 150 μL of DMSO. The absorbance of sample was measured at 490 nm using a microplate reader. Data were presented as means ± SD and the selection index (SI) was caculated as the below equation: SI = [IC_50_ (nomarl cells)/IC_50_ (cancer cells)] × 100 [34].

### Clonogenic cell survival assays

Clonogenic survival was evaluated by colony formation assays. Briefly, cell suspensions were seeded into 12-well plates (300 cells per well). After adhering overnight, cells were treated with NC or DDP for 10–14 days. Eventually, colonies were fixed with formalin and stained with 0.25% (w/v) crystal violet and colonies with more than 50 cells were included in the quantification. The colony number = number of colonies formed/number of cells seeded.

### Microscopy imaging

To examine the morphology of pyroptotic cells, H1688 or A549 cells were seeded in the 24-well plate at about 80% confluency. After treated with NC or DDP, the bright-field cell images were captured using an inverted microscope (EVOS FL Auto, Thermo Scientific, America). To examine the sub-morphology of pyroptotic cells, cells were seeded in 10 cm culture dishes for 12 h. After treated with NC or DDP, the pore-forming activity of drug-induced pyroptosis was examined by transmission electron microscopy (TEM, Hitachi-7650, Japan).

### Hoechst 33,342/PI staining

Hoechst 33,342/PI staining assay was performed according to the manufacturer’s instructions (Apoptosis and Necrosis Assay Kit, Beyotime Institute of Biotechnology, China). H688 and A549 cells were cultured in the 24-well plate (Corning Incorporated, Corning, USA) and subjected to the indicated treatments. Then hoechst 33,342 (5 μL) and PI dye (5 μL) were added and then incubated for 20 min in the dark and visualized under an inverted fluorescence microscope (EVOS FL Auto, Thermo Scientific, USA).

### Western blot (WB)

Cells or tissue samples were collected and lysed in RIPA buffer containing proteinase inhibitors (Sigma, USA) and phosphatase inhibitors (CWBio, China). The protein extracts were separated on SDS-PAGE gels, followed by electrotransfering onto polyvinylidene fluoride membranes (Sigma, USA). After blocking with 5% fat-free milk for 2 h at room temperature, the membranes were incubated with primary antibodies and subsequently incubated with secondary antibody (Thermo Scientific, MA, USA). Finally bands were scanned with Bio-Rad Imager and individual band intensity was quantified with software of ImageJ. Antibodies were used as follows: caspase 3 (CST, USA), cleaved caspase 3 (CST, USA), GSDMC (Abclonal, China), GSDMD (Abclonal, China), GSDME (Abclonal, China), PI3K (Bioss, China), p-PI3K (Bioss, China), Akt (Proteintech, China), p-Akt (CST, USA), Bcl-2 (Proteintech, China), Bax (Proteintech, China) and β-Actin (Abclonal, China).

### Lactate dehydrogenase (LDH) release assays

Cell culture supernatants were collected and the LDH activity was detected using the LDH assay kit according to the manufacturer’s protocol (Beyotime Institute of Biotechnology, China). The supernatants (120 μ L/well) were transferred into a blank 96-well plate and 60 μ L of LDH detection reagents were added to each well. The plates were then incubated for 30 min at room temperature in the dark. The absorbance was measured at 450 nm on a spectrophotometric microplate reader (BioTek, USA).

### Network pharmacology analysis

The key word “nitidine chloride” was searched in Herb (http://herb.ac.cn/) and Swiss TargetPrediction (https://pubchem.ncbi.nlm.nih.gov/) databases. After merging and deleting duplicate values, a total of 870 targets were obtained. With “lung cancer” as the key word, MalaCards (http://www.malacards.org/) and GeneCards (www.genecards.org/) potential disease target analysis platforms were utilized to search for human genes related to LC. After merging and deleting duplicate values, a total of 1160 targets were obtained. The overlapped targets between NC and LC were further analyzed by the KEGG enrichment analysis using the DAVID database (https://david.ncifcrf.gov/).

### Cellular thermal shift assay (CETSA)

CETSA was conducted using cell lysates as previously described [13]. Cell lysis buffer treated with NC or DMSO at room temperature for 1 h were collected and the cell suspension was distributed into 0.2 ml PCR tubes, with 200 µl cell suspension in each tube. The PCR tubes were heated at the designated temperature (37, 42, 47, 52, 57 and 62 °C) for 3 min. They were then removed and incubated at 4 °C immediately after heating. The precipitated proteins were separated from the soluble fraction by centrifugation and equal portions of the supernatants were loaded onto SDS-PAGE gels for WB analysis.

### Drug affinity responsive target stability (DARTS) assay

H1688 and A549 cells were lysed in 500 μL NP-40 containing freshly protease (Sigma, America) and phosphatase inhibitors (CWBio, China). The cell lysate was equally divided into two tubes and then incubated with NC (0.2 mg/mL) and an equal volume of DMSO for 1 h at room temperature, respectively. After incubation, the mixture was divided into 100 μL aliquot in tubes and digested with pronase (MCE, America) in various doses at room temperature for 30 min. Then the digestion was stopped by adding protease inhibitors, and the samples were used for WB analysis.

### Cell transfection

A small interfering RNA (siRNA) that specifically targets human caspase was designed (RiboBio, China). Si-NC or si-caspase 3 was transfected into H1688 and A549 cells using Lipo3000™ (Thermo Scientific, MA, USA) according to the manufacturer’s protocol. The medium was replaced with complete growth medium after 24 h of transfection and the cells were treated with NC.

### Animal experiment

Four-to-five-week-old BALB/c male mice were purchased and maintained in the animal experimental center of Guangxi Medical University. H1688 cells or A549 cells (approximately 1 × 10^7^) were injected subcutaneously into the right flank of BALB/c mice. Considering that NC is poorly absorbed, we treated the mice with NC by intraperitoneal injection. Once the tumors volume reached an average of 50–80 mm^3^, the mice were randomized into 4 groups (n = 5), with either vehicle control (0.1% DMSO), lower dose of NC (8 mg/kg/week), or higher dose of NC (16 mg/kg/week) twice a day respectively. As a positive control, mice were injected with DDP (8 mg/kg/week). The tumor volume (length × width × width) and body weight of the mice were monitored every 2 days. At the end of treatment, mice were sacrificed and tumors were removed, weighted and analyzed via hematoxylin and eosin staining (H&E), immunohistochemistry (IHC) and WB.

### Immunohistochemistry

After routine dewaxing and hydration, the tissue sections from mouse tumor tissue specimens of each group were boiled in a microwave oven for 10 min for antigen retrieval followed by washing three times with PBS (pH = 7.4). Then they were treated with 3% hydrogen peroxide for 25 min and washed three times with PBS (pH = 7.4). Subsequently, the tissue sections were blocked in blocking buffer containing 3% BSA and incubated with Ki67, p-PI3K, cleaved caspase 3 and GSDME‐N antibodies overnight at 4 °C followed by incubating with the secondary antibody and DAB. The sections were counterstained using hematoxylin for 3 min and washed under tap water. The images were captured by the inverted microscope (EVOS FL Auto, Thermo Scientific, USA).

### Statistical analysis

Statistical analysis was performed with GraphPad Prism 9.0 software. In all experiments, comparisons between two groups were based on unpaired student’s t-test and multiple group comparisons were performed using one-way ANOVA followed by least significant difference (LSD) post hoc test. *P* < 0.05 was considered statistically significant. The values of the results were representative in terms of the mean ± standard deviation (SD).

## Results

### NC inhibites cell proliferation in human LC cells.

Firstly, an immortalized human normal lung epithelial cells (BEAS-2B) and 11 human LC cells lines (NCI-H1688, NCI-H196, NCI-H209, NCI-H446, NCI-H226, SK-MES-1, A549, NCI-H1299, NCI-HCC827, NCI-H1975 and PC9) were used to investigate the anti-cancer effects of NC in LC and DDP was used as a positive drug. As shown in Fig. 1B, NC inhibited the cell growth of 11 LC cell lines in a dose-dependent manner (The IC_50_ value and SI of NC for 12 cell lines were listed in Table 1). Due to the higher SI of NC in H1688 and A549 cells, we selected these two cell lines to further investigate. By MTT and clonogenic cell survival assays, we observed that NC treatment significantly suppressed the cell viability of H1688 and A549 cells in a time-dependent manner, and inhibited their clonal proliferation ability (Fig. 1C, D). Collectively, these results suggested that NC exhibited a well anti-LC activity in vitro.

**Table 1:** IC_50_ of nitidine chloride or cisplatin on human bronchial epithelial normal cells and 11 lung cancer cells for 48 h

Cell lines		IC_50_ (μmol/L)of DDP	IC_50_ (μmol/L) of NC	SI (Selectivity index) of NC
Nomal lung cell	BEAS-2B	30.451 ± 1.083	2.366 ± 0.061	–
SCLC	NCI-H1688	16.933 ± 1.509	1.520 ± 0.076	1.56
	NCI-H446	16.724 ± 1.696	1.987 ± 0.208	1.2
	NCI-H209	35.021 ± 1.85	3.261 ± 0.093	0.75
	NCI-H196	16.217 ± 1.734	4.649 ± 0.571	0.51
LUSC	SK-MES-1	14.652 ± 1.442	6.703 ± 0.025	0.36
	NCI-H226	31.816 ± 0.513	3.813 ± 0.113	0.62
LUAD	A549	13.677 ± 0.511	0.706 ± 0.033	3.42
	NCI-HCC827	24.811 ± 2.3	3.053 ± 0.178	0.77
	NCI-H1299 NCI-H1975 PC9	27.207 ± 0.82 11.770 ± 0.398 11.876 ± 0.184	4.791 ± 1.042 5.072 ± 0.183 5.54 ± 0.547	0.6 0.47 0.44

(Means ± standard deviation)

### NC changes permeability of plasma membrane and induces pyroptotic-like morphology

**Fig. 2:**
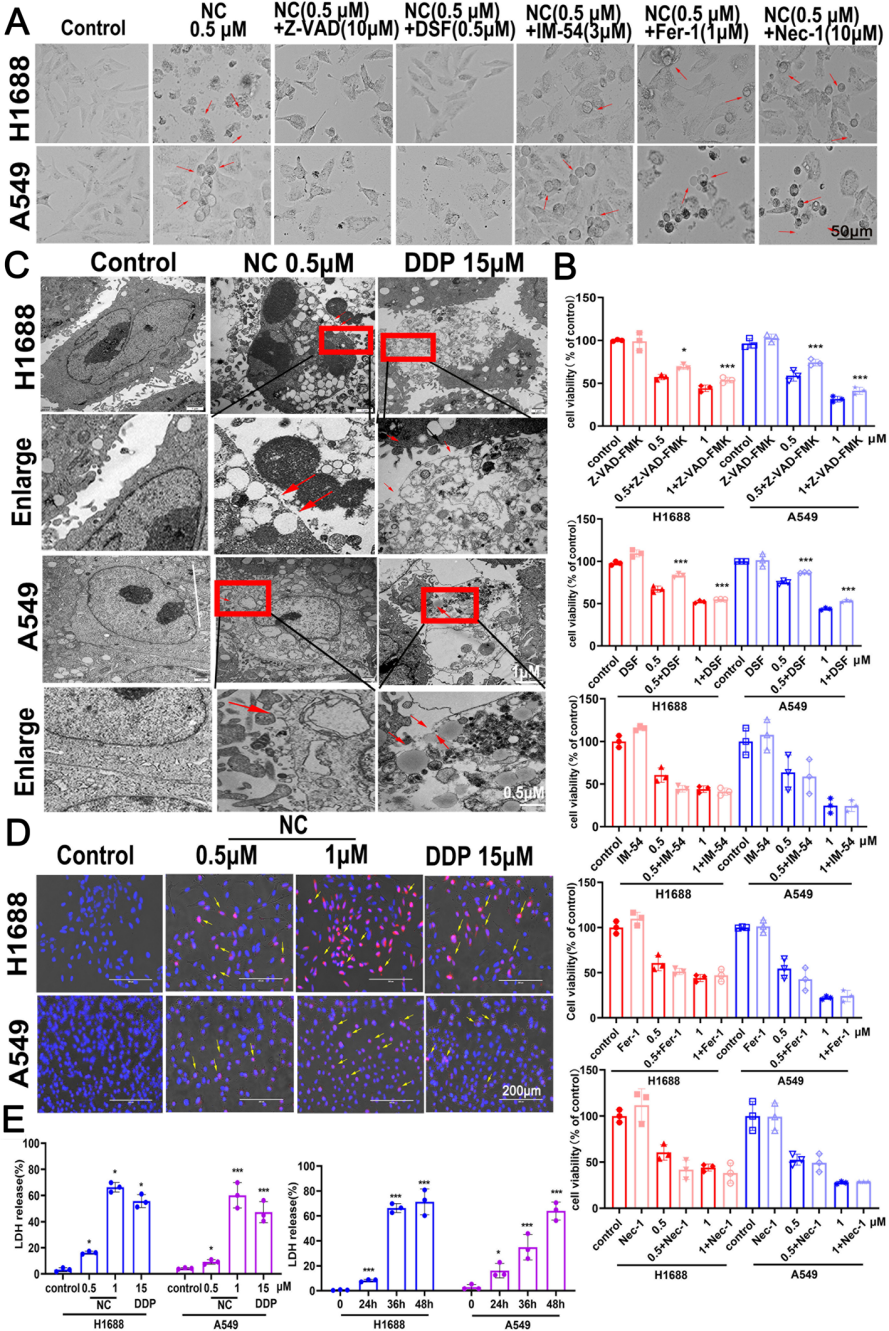
**A** and **B** H1688 and A549 cells were treated with NC with or without Z-VAD-FMK, DSF, IM-54, Fer-1, Nec-1 respectively. After 48 h of treatment, the inhibition of growth was assayed. **C** H1688 and A549 cells were treated with NC (0.5 μm) or DDP (15 μm) for 48 h and microscopic imaging was captured. Arrowheads indicated ballooned cell membrane characteristic of pyroptotic cells. **D** H1688 and A549 cells were treated with 0.5 and 1 μm NC or 15 μm DDP for 48 h, hoechst 33,342 and PI dye were added and visualized under an inverted fluorescence microscope. Yellow arrowheads indicated the large bubbles emerging from the plasma membrane. **E** H1688 and A549 cells were treated with NC or DDP at indicated concentrations for 48 h, the release of LDH in cell supernatants were analyzed using LDH assay kit and expressed as mean ± SD (n = 3). *P < 0.05, **P < 0.01, ***P < 0.001 *vs* control

Five inhibitors of cell death were utilized to determine the role of cell death induced by NC in LC cells. As shown in Fig. 2A, B, pretreatment of IM-54 (a necrosis inhibitor), fer-1(a potent inhibitor of ferroptosis) or nec-1 (a potent inhibitor of necroptosis) did not protect against NC-inducing cell death in H1688 and A549 cells. Interestingly, the decrease of cell viablity induced by NC treatment was partially impaired by pretreatment with Z-VAD-FMK (an apoptotic inhibitor) or DSF (a pyoptosis inhibitor), indicating that NC may trigger apoptotic or pyoptotic cell death in LC cells. As previous studies have revealed that apoptosis is involved in NC-inducing cell death in LC cells [10, 14, 15], we focused primarily on pyroptosis in the following experiments. The results of hochest/PI staining showed that a significant lytic cell death was observed in H1688 or A549 cells treated by NC. Meanwhile, cells treated with NC displayed a characteristic morphology change of pyroptosis as represented by a balloon-like bubbles, a typical feature to distinguish pyroptosis from apoptosis (Fig. 2D). In addition, transmission electron microscopy (TEM) results showed multiple pores were formed in the membranes of NC-treated in H1688 and A549 cells (Fig. 2C). The release of LDH were elevated in the supernatant of H1688 and A549 cells treated by NC in a dose-dependent and time-dependent manners, which suggested cells undergoing pyroptosis (Fig. 2E).

### NC elicits caspase-3/GSDME-dependent pyroptosis in LC cells

**Fig. 3:**
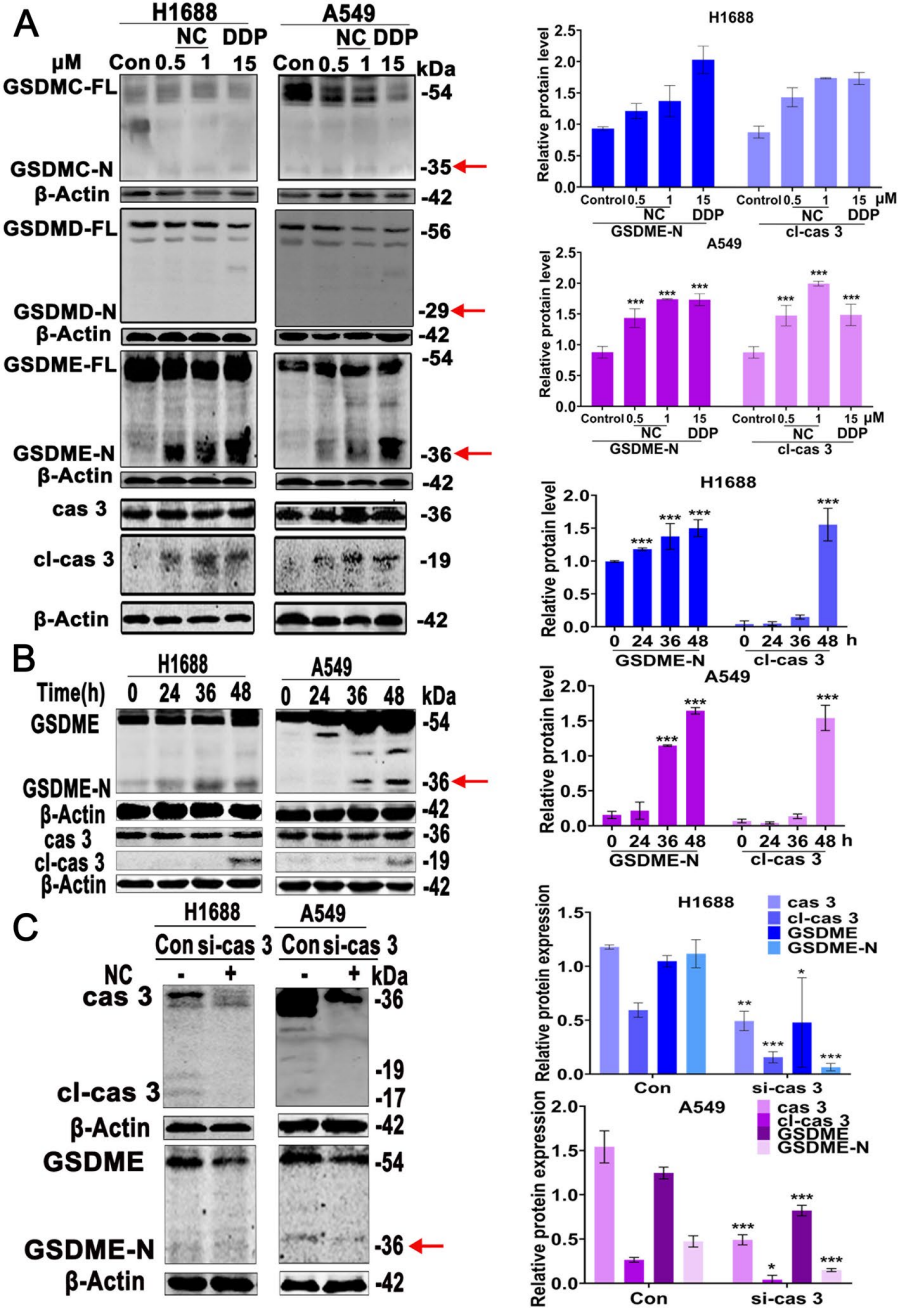
**A** H1688 and A549 cells were treated with NC (0.5 and 1 μm) or DDP (15 μm) for 48 h respectively. Total cellular extracts were prepared and subjected to WB analysis using antibodies against GSDMC, GSDMD, GSDME, cas 3, cl-cas 3 and β-Actin. **B** H1688 and A549 cells were treated with NC (0.5 μM) for 24, 36 and 48 h, total cellular extracts were prepared and subjected to WB analysis using antibodies against cas 3, cl-cas 3, GSDME and β-Actin. **C** H1688 or A549 cells were transfected with siRNA targeting caspase 3 (si-cas 3) or siRNA (control) and then treated with NC for 48 h. Protein levels are expressed as mean ± SD (n = 3). *P < 0.05, **P < 0.01, ***P < 0.001 *vs.* control. *P < 0.05, **P < 0.01, ***P < 0.001 *vs* control

GSDMC, GSDMD and GSDME have been reported to trigger pyroptosis via their pore-forming activities [16, 17], which was closely related to their protein expressions [18]. Therefore, we detected the protein expression of GSDMC, GSDMD and GSDME in BEAS-2B and 11 LC cell lines (Additional file 1: Fig. S1). As shown in Additional file 1: Fig. S1D, the expressions of GSDMC, GSDMD and GSDME were markedly higher in H1688 and A549 cells compared with those in BEAS-2B cells and other 9 LC cell lines, supporting our in vitro results that H1688 and A549 cells were more sensitive to NC treatment. Furthermore, we found that NC induced the expression of cleaved GSDME protein instead of GSDMC or GSDMD in a dose-dependent (Fig. 3A). GSDME was identified as the substrate of caspase 3 and the cleaved GSDME can release the N-terminal domain of GSDME to pore the plasma membrane and thereby induce pyroptosis [18]. Our results demonstrated that the cleaved caspase 3 was robustly activated in a dose-dependent after treatment of NC in H1688 and A549 cells. Moreover, the activations of the cleavage of caspase 3 and GSDME-N were increased in a time-dependent way (Fig. 3B). Additionally, the results yield from knockdown of caspase 3 showed that GSDME-N in si-caspase 3 cells were obviously obstructed after NC treatment (Fig. 3C and Additional file 1: Fig. S2).

### NC directly targets PI3K and inhibit PI3K/Akt pathway in LC cells

**Fig. 4:**
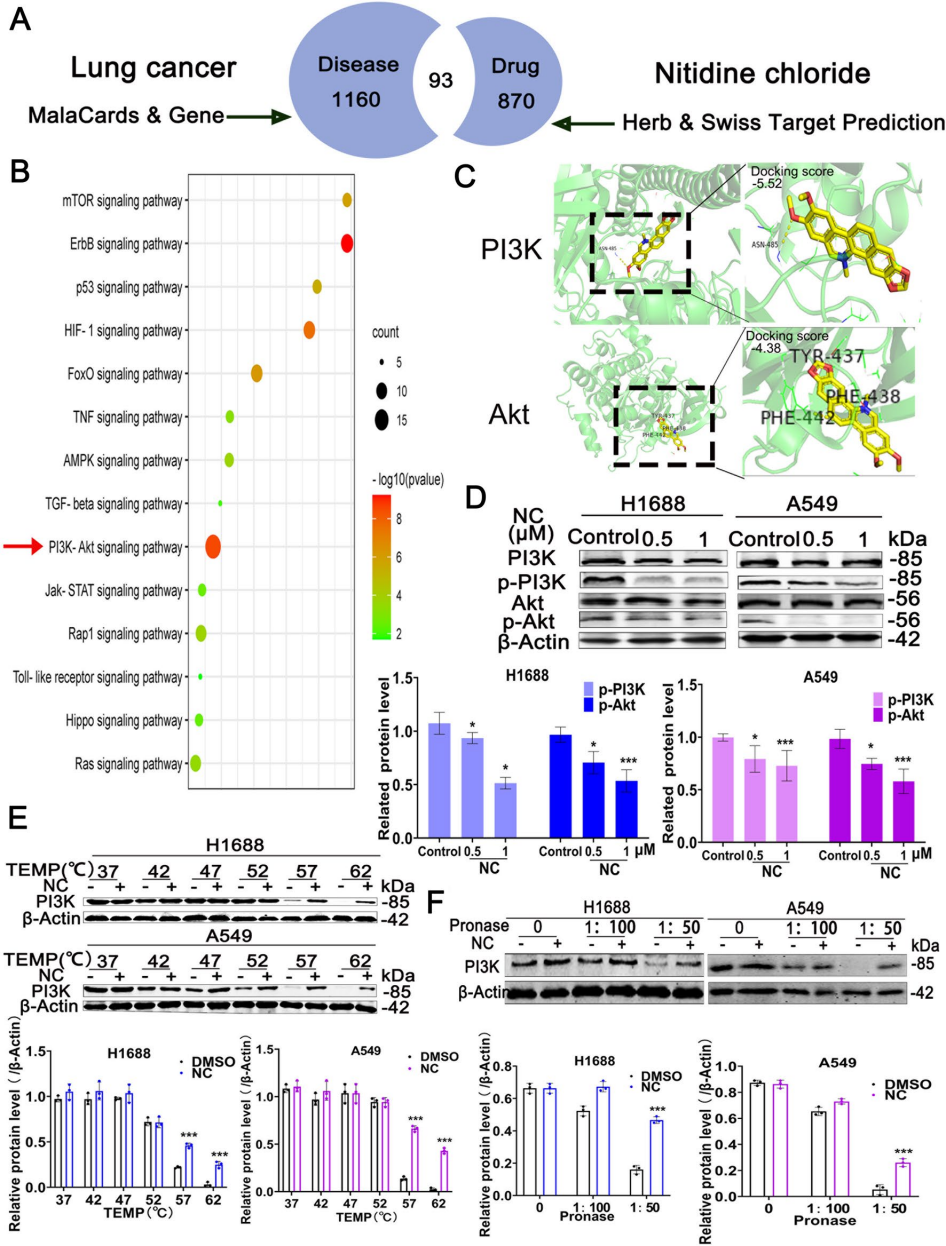
**A** Veen diagram of common gene targets of lung cancer and nitidine chloride. **B** Signaling pathways enriched terms in KEGG pathways. **C** Molecular docking analysis of NC targeting PI3K or Akt. **D** H1688 and A549 cells were treated with NC for 48 h, total cellular extracts were prepared and subjected to WB analysis using PI3K, p-PI3K, Akt, p-Akt and β-Actin antibodies. **E** CETSA was performed with cell lysates of H1688 or A549 cells that were treated with DMSO or NC with an increasing melting temperature (37 ℃-62 ℃, interval temperature: 5 ℃). **F** WB analysis of DARTS sample further revealed the increased stabilization of PI3K during proteolysis process in H1688 or A549 cells. Protein levels are expressed as mean ± SD (n = 3). *P < 0.05, **P < 0.01, ***P < 0.001 *vs* control

To investigate the underlying mechanism by which NC induced pyropotosis, the network pharmacology analysis was performed. As shown in Fig. 4A, 93 targets were obtained after intersecting analysis of predicted targets of LC and NC. The details of common target genes are shown in Additional file 1: Table S1. Further KEGG analysis using DAVID database, 14 significant pathways such as PI3K/AKT signaling pathway, ErBb signaling pathway and p53 signaling pathway and so forth are enriched (Fig. 4B). Among them, PI3K/AKT signaling pathway plays an important role in pyroptosis. Continuous activation of PI3K/AKT is able to inhibit pyroptosis while blocking of this pathway triggers pyroptosis [19]. Notably, as shown in Fig. 4C, our analysis using molecular docking predicted that there were a close interaction between NC and PI3K (interacting site: ASN-485). By WB assay, we found NC significantly inhibited the protein levels of p-PI3K and p-Akt in a dose-dependent manner with negligible effects on their total protein (Fig. 4D). To further confirm the activity of NC binding to PI3K protein, CETSA and DARTs, two classic methods to detect the binding of a drug to a target protein [20, 21], were employed. Compared to the DMSO group, NC treatment (0.2 mg/mL) increased the thermal stability of PI3K at 57 ºC and 62 ºC (Fig. 4E). As shown in Fig. 4F, WB analysis of DARTS sample further revealed the increased stabilization of PI3K during the proteolysis process when the cell lysis of H1688 and A549 cells treated with NC at 0.2 mg/mL (1:50). Collectively, these data indicated that NC could inhibit PI3K/Akt pathway by directly targeting PI3K in LC cells.

### The pyroptosis induced by NC was dependent on the suppression of PI3K/Akt pathway

**Fig. 5:**
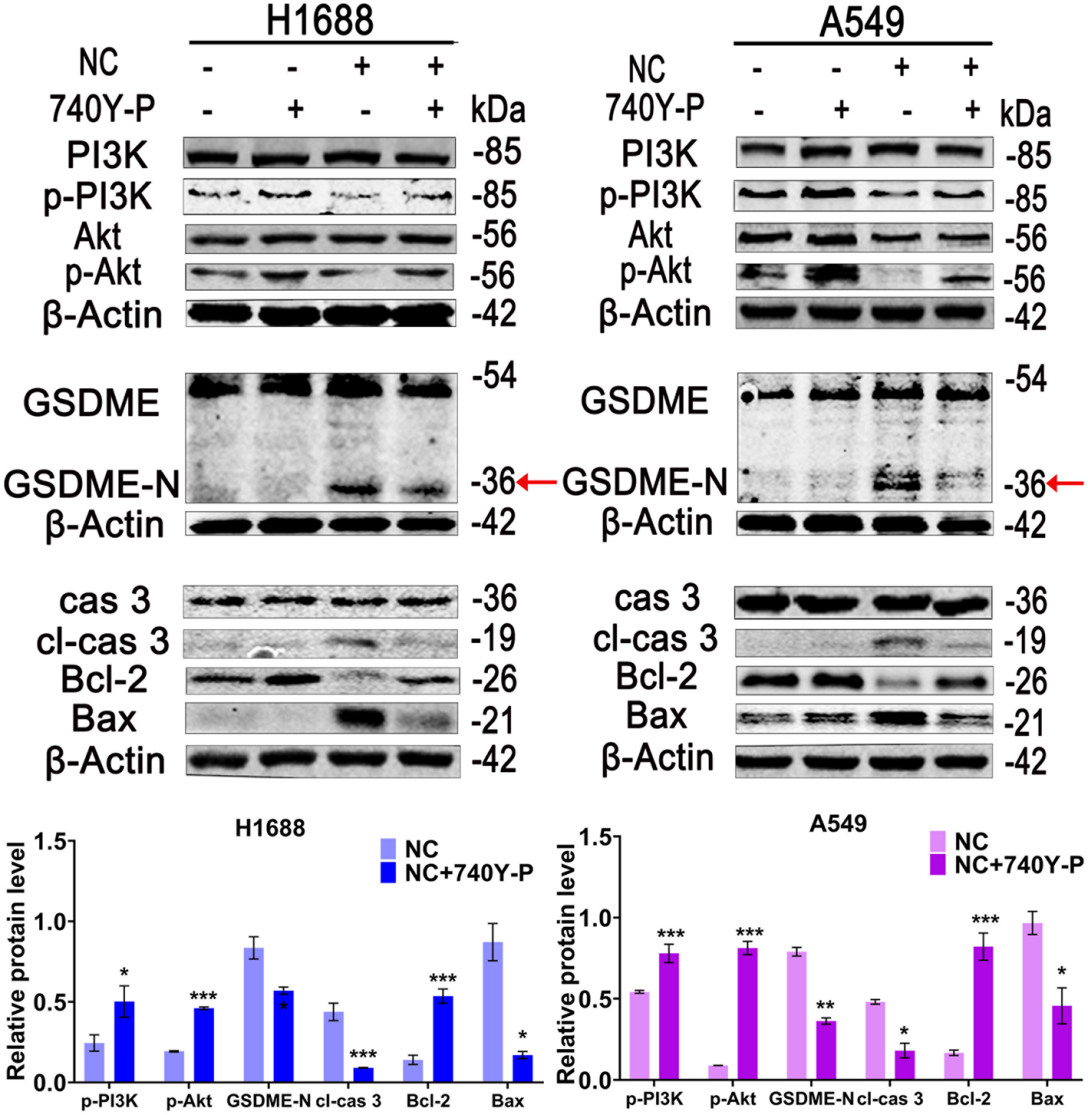
The cells were pre-treated with 20 μm 740Y-P or 0.5 μm NC alone or combined with 20 μm 740Y-P for 48 h. WB analyses were performed to detected the protein levels of PI3K, p-PI3K, Akt, p-Akt, GSDME, cas 3, cl-cas 3, Bcl-2, Bax and β-Actin. Protein levels were expressed as mean ± SD (n = 3). *P < 0.05, **P < 0.01, ***P < 0.001 *vs* control

It has been reported that the blockage of PI3K/Akt pathway activate the cleavage of caspase 3 protein, which induced GSDME-N formation, triggering pyropotosis in LC cells [22]. To further explore the role of PI3K/Akt signaling pathway in the pyroptosis induced by NC in LC cells, 740Y-P (an activator of PI3K) was used. Compared with NC group, 740Y-P significantly reduced the release of cleaved caspase 3 and GSDME-N while boosted the phosphorylation of PI3K and Akt (Fig. 5). These results demonstrated that NC-induced pyroptosis in LC cells was mediated by PI3K/Akt signaling pathway.

### NC inhibites the tumor growth in Xenograft Mouse Model

**Fig. 6:**
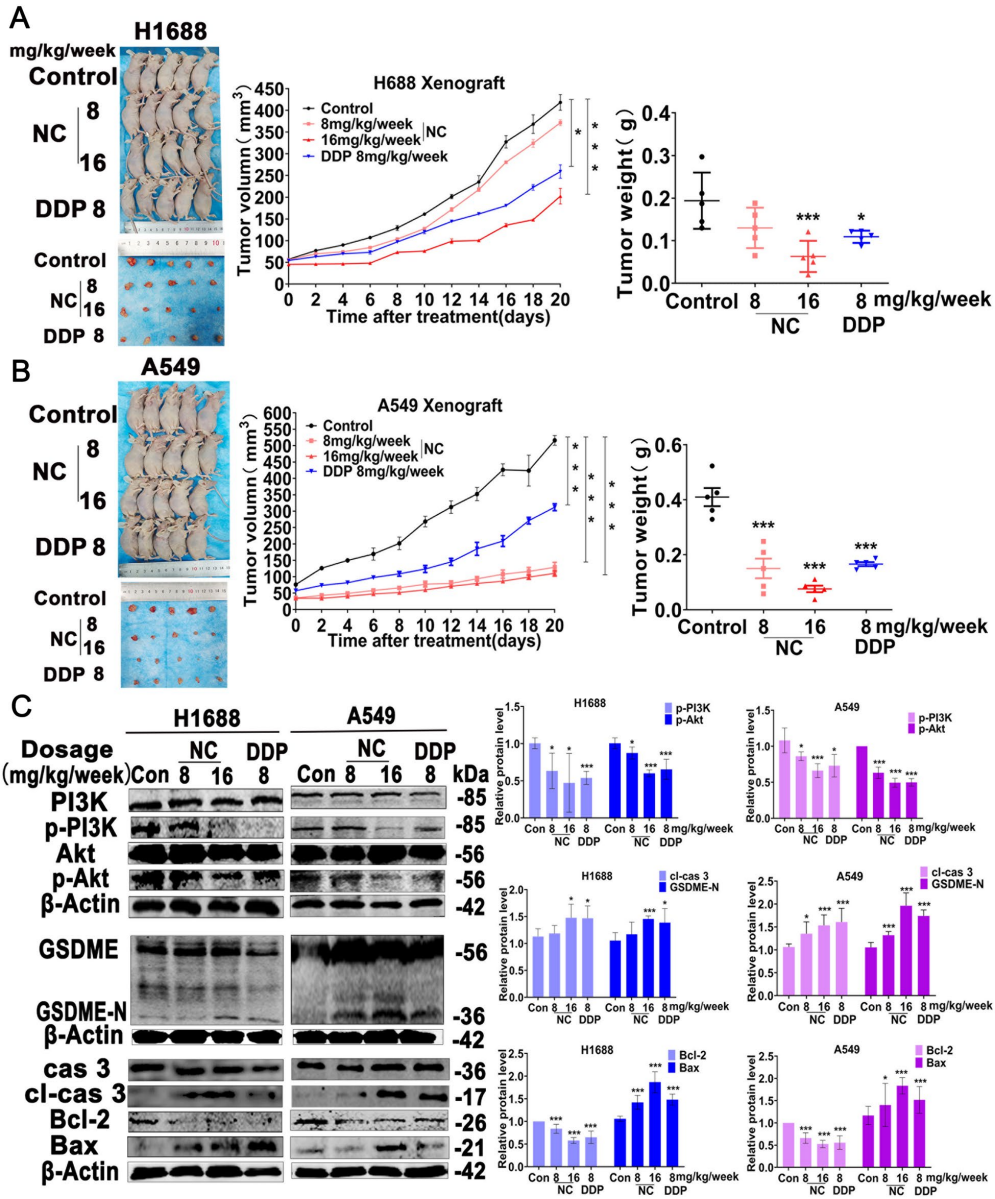
**A** and **B** Representative photographs, tumor volume measurement and tumor weight in H1688 or A549 xenograft mouse model at 21 d after NC treatment (n = 5). **C** The protein expression of PI3K, p-PI3K, Akt, p-Akt, GSDME, cas 3, cl-cas 3, Bcl-2, Bax and β-Actin in treated tumor tissues. Protein levels were expressed as mean ± SD (n = 5). *P < 0.01, **P < 0.05, ***P < 0.001 *vs* control

**Fig. 7:**
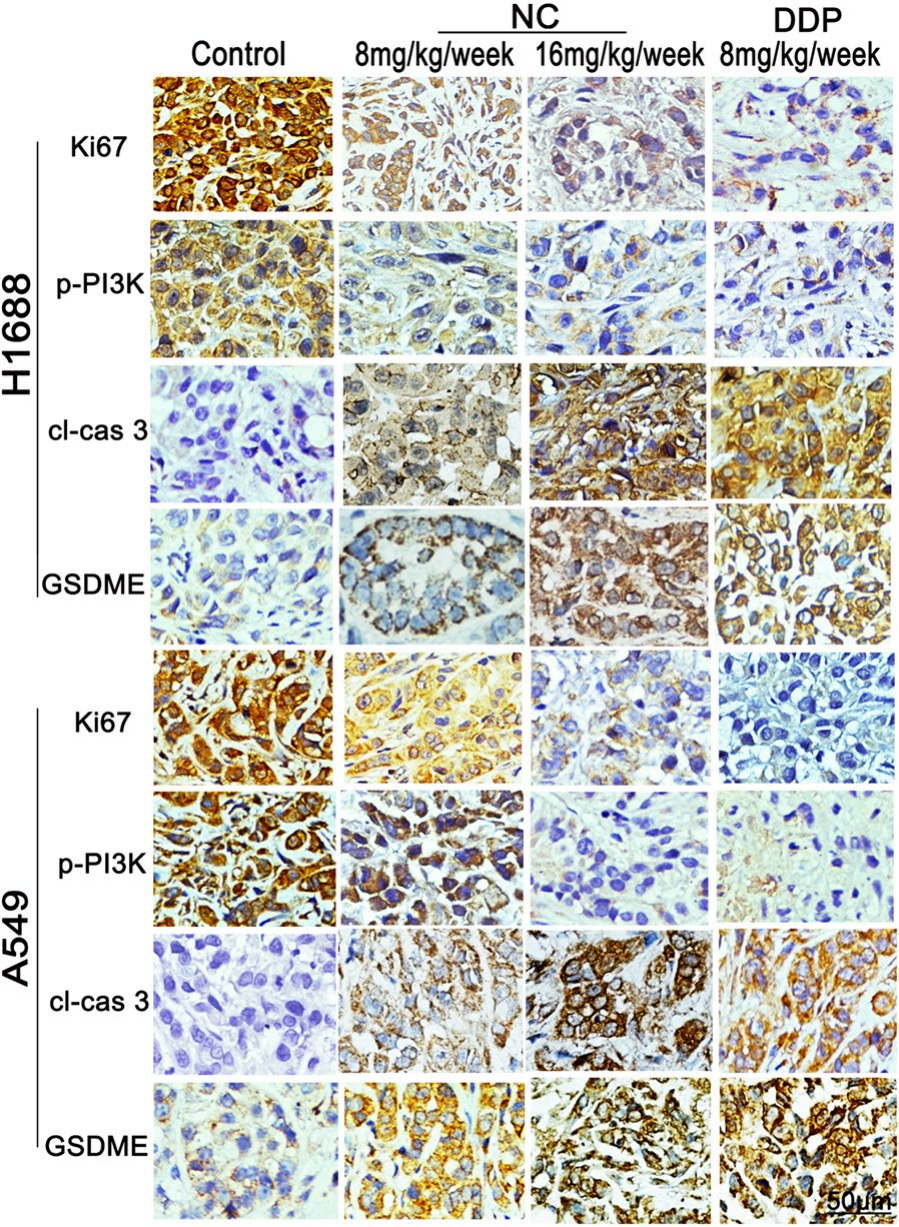
The protein expression of Ki67, p-PI3K, cl-cas 3 and GSDME in H1688 or A549 xenograft tumors were detected by immunohistochemistry, scale bar = 50 μm

We further investigated the inhibitory effects of NC on LC in a xenograft mice model. As shown in Additional file 1: Fig. S3, S4, there was no significant difference between the average body weight of NC-treated and control mice. Consistently, H&E staining revealed that no morphological change was observed in the hearts, livers, spleens, lungs and kidneys after NC treatment, suggesting NC had no obvious toxicity to mice. The volume of the xenograft tumors treated with NC were remarkably smaller than that in control group (Fig. 6A, B). Moreover, the results yield from WB in tumor tissues showed that NC treatment up-regulated the protein levels of cleaved caspase 3 and N-GSDME, and inhibited the phosphorylation of p-PI3K and p-Akt in a dose-dependent manner (Fig. 6C). In addition, we also investigated the expression of Ki-67, p-PI3K, cleaved caspase-3 and GSDME by IHC. As shown in Fig. 7, the expression of Ki-67 was significantly decreased after NC treatment, suggesting that NC could inhibit the growth of LC. Meanwhile, NC suppressed the expression of p-PI3K and elevated the expression of cleaved caspase 3 and GSDME-N. These results suggested that NC induced the pyroptosis of LC cell in vivo by inhibiting PI3K/Akt pathway.

## Discussion

LC is responsible for the majority of cancer deaths worldwide [23]. Despite the improved surveillance programs for LC globally have achieved, the overall 5-year survival remains poor (20%) [24]. Chemotherapy drug such as DDP is the mainstay treatment for advanced LC, but they only provides limited survival benefit for patients [25, 26]. In our study, we found NC significantly inhibited the growth of H1688 and A549 cells in vitro and in vivo*,* consistent with the previous report that of NC had a potential therapeutic for LC [8].

Resisting cell death is one of hallmarks of cancer and is necessary for tumor cells evading death and promote the tumor development and progression [27]. Therefore, specifically inducing cell death with drugs is acknowledged as a promising anti-tumor strategy. Natural compounds are an abundant source for the discovery of anti-cancer agents such as vincristine, vinblastine, paclitaxel, curcumin, colchicine and lycopene [28, 29]. Lots of studies has reported NC exerted good efficacies for a variety of cancer by inducing apoptosis and autophagy [14, 15, 30]. In the present study, we observed that pretreatment with the apoptosis inhibitor (Z-VAD-FMK) or pyroptosis inhibitor (DSF) partly increased the cell viability after NC treatment rather than IM-54, nec-1, or fer-1. Thus, it could be deduced that pyroptosis might be also involved in NC-induced cell death in LC cells apart from apoptosis. By a series of additional experiments including hoechst 33,342/PI staining and TEM, we confirmed that NC induced pyropotosis-like morphology changes. Subsequently, to determine which type of gasdermin was responsible for executing pyroptosis after NC treatment, we tested the protein expression of cleaved in LC cells. Our results showed that NC specifically activated the cleavage of GSDME, without obvious effects on GSDMC or GSDMD cleavage. It has been proved that cleaved caspase 3 is necessary for cleaving GSDME to produce active N-terminal domain to initiate pyroptosis [18]. Hence, the protein levels of cleaved caspase 3 were explored evidently increased in NC-treated H1688 and A549 cells. Furthermore, knockdown of caspase 3 with siRNA significantly inhibited the GSDME-N formation induced by NC.

The PI3K/Akt signaling pathway mainly regulates cell proliferation, inflammation, survival and metastasis to maintain the biological properties of malignant cells [31]. Recently, it has been suggested that inhibition of this pathway triggered cell pyroptosis. By network pharmacology analysis, we revealed PI3K/Akt signaling pathway enriched. Moreover, our results of in silico analysis using molecular docking indicated that NC was capable of binding to PI3K protein through hydrogen bonds at ASN-485. Based on these findings, we looked at PI3K/Akt pathway to explore the underlying mechanism by which NC induced the pyroptosis of LC cells. Our data showed that NC exhibited a dose-dependent inhibition in PI3K/Akt pathway, as evidenced by decreased phosphorylation of PI3K and AKT, in line with the earlier findings that NC regulated PI3K/Akt pathway in different types of cancer cells [32]. Besides, 740 Y-P, a classic activator of PI3K, was able to inhibit the activation of cleaved caspase 3 and GSDME-N induced by NC treatment. Simultaneously, we found NC could hinder PI3K/Akt pathway and triggered the pyroptosis of LC tumors in mouse model of subcutaneous implantation. However, it remains unclear how NC directly targets PI3K and their interaction need to be further investigated by surface plasmon resonance (SPR) or biolayer interferometry (BLI) technology.

## Conclusions

**Fig. 8:**
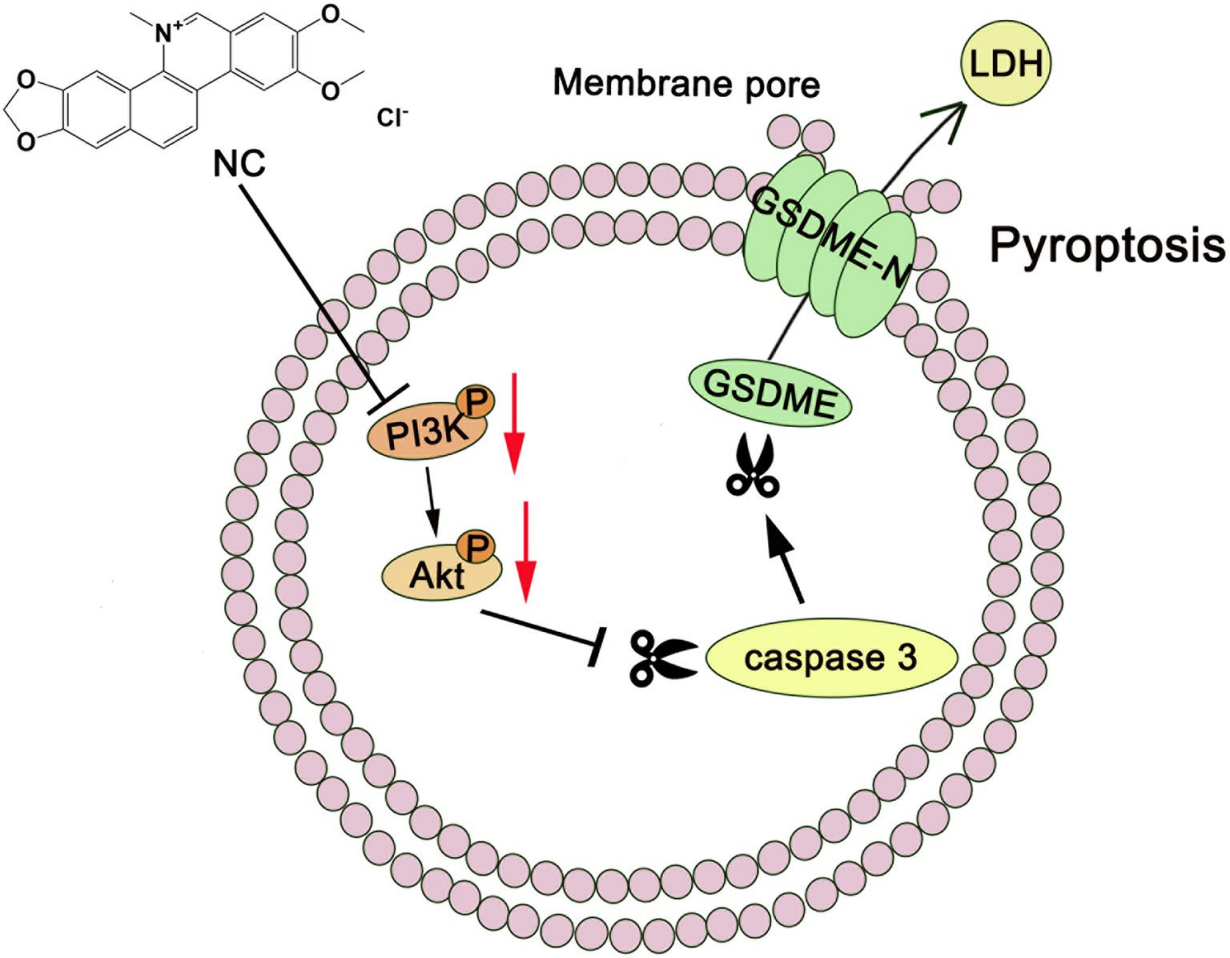
Schematic diagram of the mechanism of NC-induced pyroptosis in LC cells

In summary, NC may act as a potential natural PI3K inhibitor to induce pyroptosis in lung cancer, providing a new insight for the development of NC in the treatment of lung cancer (Fig. 8).

## Supplementary Information

**Additional file 1: Table S1** The overlapped targets between NC and lung cancer. **Fig.** **S1** **A–C **GSDMC, GSDMD and GSDME protein expression were analyzed by Western blotting. **D** WB analyses of GSDMC, GSDMD and GSDME in lung cancer cell lines and BEAS-2B cell line. Protein levels are expressed as mean ± SD (n=3). **Fig.** **S2** H1688 or A549 cells were transfected with siRNA targeting caspase 3 (siRNA-cas3-1/2/3) or control siRNA for 24 h. **Fig. S3** **A** Body weight of H1688 xenograft mice during the 20 days’ treament (presented as mean ± SD, n=5). **B** HE of hearts, livers, spleens, lungs, kidneys and tumors at the end of experiment. Scale bar=50 μm. **Fig. S4** **A** Body weight of A549 xenograft mice during the 20 days’ treament (presented as mean±SD, n=5). **B** HE of hearts, livers, spleens, lungs, kidneys and tumors at the end of experiment. Scale bar=50 μm.

## Data Availability

All data generated or analyzed during this study are included in this published article.
