# Supplementary material for: Nitidine chloride induces caspase 3/GSDME-dependent pyroptosis by inhibting PI3K/Akt pathway in lung cancer
Source: Chin Med. 2022 Sep 29;17:115. doi: 10.1186/s13020-022-00671-y (PMC9524076; doi:10.1186/s13020-022-00671-y)
Supplement: Supplementary file 1 — Additional file 1: Table S1 The overlapped targets between NC and lung cancer. Fig. S1 A–C GSDMC, GSDMD and GSDME protein expression were analyzed by Western blotting. D WB analyses of GSDMC, GSDMD and GSDME in lung cancer cell lines and BEAS-2B cell line. Protein levels are expressed as mean ± SD (n=3). Fig. S2 H1688 or A549 cells were transfected with siRNA targeting caspase 3 (siRNA-cas3-1/2/3) or control siRNA for 24 h. Fig. S3 A Body weight of H1688 xenograft mice during the 20 days’ treament (presented as mean ± SD, n=5). B HE of hearts, livers, spleens, lungs, kidneys and tumors at the end of experiment. Scale bar=50 μm. Fig. S4 A Body weight of A549 xenograft mice during the 20 days’ treament (presented as mean±SD, n=5). B HE of hearts, livers, spleens, lungs, kidneys and tumors at the end of experiment. Scale bar=50 μm. [file 13020_2022_671_MOESM1_ESM.docx]

Supplementary Materials for

**Nitidine chloride induces caspase 3/GSDME-dependent pyroptosis by inhibting PI3K/Akt pathway in lung cancer**

**Table S1 The overlapped targets between NC and lung cancer.**

| NO. Gene Name | | NO. Gene Name | | | NO. Gene Name | |  |
| --- | --- | --- | --- | --- | --- | --- | --- |
| 1 | NMB | 36 | IGFBP3 | | 71 | SERPINB5 | |
| 2 | SAT1 | 37 | MSMB | | 72 | XPC | |
| 3 | TFPI2 | 38 | YAP1 | | 73 | GNAQ | |
| 4 | PRKAA1 | 39 | MUC16 | | 74 | DAPK1 | |
| 5 | AKR1B10 | 40 | CYP1B1 | | 75 | GADD45A | |
| 6 | FGFR3 | 41 | SETD2 | | 76 | PIK3CB | |
| 7 | ESR1 | 42 | HERC2 | | 77 | ACTA2 | |
| 8 | LINC00342 | 43 | SNAI2 | | 78 | HPGD | |
| 9 | MAP3K8 | 44 | PRKCA | | 79 | CA9 | |
| 10 | BRIP1 | 45 | SERPINA3 | | 80 | FSCN1 | |
| 11 | NCOR1 | 46 | BARD1 | | 81 | VDR | |
| 12 | CDKN1A | 47 | DPYD | | 82 | GRB2 | |
| 13 | JAK2 | 48 | EHBP1 | | 83 | WRN | |
| 14 | SOX9 | 49 | AURKA | | 84 | GDF15 | |
| 15 | RB1 | 50 | TOP1 | | 85 | TCF7L2 | |
| 16 | CYP1A1 | 51 | CHEK2 | | 86 | ASCL1 | |
| 17 | FAS | 52 | CEACAM6 | | 87 | PARN | |
| 18 | BCL2 | 53 | CREBBP | | 88 | MYB | |
| 19 | IGF1R | 54 | PIK3CA | | 89 | NAT1 | |
| 20 | HNRNPA2B1 | 55 | IGF2R | | 90 | GSK3B | |
| 21 | SERPINA1 | 56 | MTOR | | 91 | BCL6 | |
| 22 | CDC73 | 57 | PLA2G2A | | 92 | TAP1 | |
| 23 | PGR | 58 | PTGS2 | | 93 | KDM4C | |
| 24 | MAD1L1 | 59 | THBS1 |  | | | |
| 25 | BMPR1A | 60 | PIK3CG |  | | | |
| 26 | PLAU | 61 | RPS6KB1 |  | | | |
| 27 | RET | 62 | AREG |  | | | |
| 28 | DDB2 | 63 | CYP2D6 |  | | | |
| 29 | PIK3R1 | 64 | EPHA2 |  | | | |
| 30 | CD44 | 65 | DHFR |  | | | |
| 31 | PTK2 | 66 | HDAC1 |  | | | |
| 32 | MIR22 | 67 | CADM1 |  | | | |
| 33 | FBN1 | 68 | BMP6 |  | | | |
| 34 | CXCL12 | 69 | MYCL |  | | | |
| 35 | ABCC1 | 70 | RS1 |  | | | |

**Fig. S1 A-C** GSDMC, GSDMD and GSDME protein expression were analyzed by WB. **D** WB analyses of GSDMC, GSDMD and GSDME in lung cancer cell lines and BEAS-2B cell line. Protein levels are expressed as mean ± SD (n=3).

**Fig. S2** H1688 or A549 cells were transfected with siRNA targeting caspase 3 (siRNA-cas3-1/2/3) or control siRNA for 24 h.

**Fig. S3 A** Body weight of H1688 xenograft mice during the 20 days’ treament (presented as mean ± SD, n=5). **B** HE of hearts, livers, spleens, lungs, kidneys and tumors at the end of experiment. Scale bar=50 μm.

**Fig. S4 A** Body weight of A549 xenograft mice during the 20 days’ treament (presented as mean ± SD, n=5). **B** HE of hearts, livers, spleens, lungs, kidneys and tumors at the end of experiment. Scale bar=50 μm.
